# Uncovering the knowledge about systemic amyloidosis relevant to the rheumatologists

**DOI:** 10.1186/s42358-024-00399-3

**Published:** 2024-09-16

**Authors:** Ivanio Alves Pereira, Nilton Salles Rosa Neto, Renan Rodrigues Neves Ribeiro do Nascimento, Eutilia Andrade Medeiros Freire, Fabricio de Souza Neves, Blanca Elena Rios Gomes Bica, Frederico Augusto Gurgel Pinheiro, Sandro Félix Perazzio, Rafael Alves Cordeiro, Henrique Ayres Mayrink Giardini, Valderilio Feijo Azevedo, Flavio Roberto Sztajnbok

**Affiliations:** 1 Rheumatology, Universidade do Sul de Santa Catarina, Av. Rio Branco 448 sala 306 Florianópolis-SC, Florianópolis, CEP 88015200 Brazil; 2 2Rheumatologist, Centro de Doenças Raras e da Imunidade, Hospital Nove de Julho, São Paulo and Professor of Rheumatology, Universidade Santo Amaro, São Paulo, Brazil; 3 Division of Rheumatology, Universidade Federal de São Paulo (UNIFESP), Sao Paulo, Brazil; 4 Rheumatology, Universidade Federal da Paraíba (UFB), Paraiba, Brazil; 5 Rheumatology, Universidade Federal de Santa Catarina (UFSC), Santa Catarina, Brazil; 6 Pediatric Rheumatology, Universidade Federal do Rio de Janeiro, Rio de Janeiro, Brazil; 7 Division of Rheumatology, Hospital das Clínicas da Faculdade de Medicina da Universidade de São Paulo (HC-FMUSP), Sao Paulo, Brazil; 8 Rheumatology, Universidade Federal do Paraná (UFP), Parana, Brazil; 9 Division of Pediatric Rheumatology, Universidade Federal do Rio de Janeiro (UFRJ), Rio de Janeiro, Brazil

**Keywords:** Amyloidosis, Transthyretin, Light chains, Rare diseases, Orphan diseases

## Abstract

Amyloidosis is a localized or systemic disease caused by deposition of proteins in the extracellular space of various organs and tissues. As part of the disease, proteins that were originally soluble misfold and acquire a fibrillar conformation that renders them insoluble and resistant to proteolysis. Systemic amyloidosis is a rare, often underdiagnosed condition. In recent years, the incidence of newly diagnosed cases of amyloidosis has been increasing in association with the aging of the population and greater access to diagnostic tests. From a clinical perspective, systemic amyloidosis is frequently associated with involvement of the kidneys (causing nephrotic syndrome), heart (cardiac failure and arrhythmia), and peripheral nervous system (sensorimotor polyneuropathy and autonomic dysfunction). This condition is important to the rheumatologist for several reasons, such as its systemic involvement that mimics autoimmune rheumatic diseases, its musculoskeletal manifestations, which when recognized can allow the diagnosis of amyloidosis, and also because reactive or secondary AA amyloidosis is a complication of rheumatic inflammatory diseases. The treatment of amyloidosis depends on the type of amyloid protein involved. Early recognition of this rare disease is fundamental for improved clinical outcomes.

## Introduction

Amyloidosis is a group of diseases with a diverse presentation that occurs due to excessive buildup of protein aggregates in the extracellular space of different organs and tissues.

The term “amyloid” was coined in 1854 by Rudolph Virchow, who described macroscopic tissue changes identified with iodine-containing dyes. [1] In amyloidosis, proteins that are originally soluble misfold and acquire a fibrillar conformation, becoming insoluble and resistant to proteolysis. [2, 3] The mechanisms behind the misfolding process in amyloidosis have not been fully elucidated, but factors determining its occurrence include hereditary mutations in the variable region of light chains, proliferation of clonal plasma cells producing immunoglobulin fragments, and aging. [2–4] More than 40 types of proteins with the potential to become amyloid are currently recognized, some causing systemic amyloidosis and others causing only localized forms of the disease. [5]

On histopathology, amyloid fibrils stain with Congo red and appear birefringent with an apple-green color under polarized light. [2, 3, 5]

Systemic amyloidosis is generally considered rare; its nonspecific clinical manifestations hinder this disease from being recognized and diagnosed early or even during an individual’s entire life. The absence of an early diagnosis prevents adequate treatment, which contributes to an increased mortality in amyloidosis. [2, 4]

In the present review, we will highlight the most common amyloid proteins associated with amyloidosis in clinical practice, i.e., those originating from light chain immunoglobulins, transthyretin, amyloid protein A, and beta2-microglobulin. [2, 3]

The nominal classification of amyloidosis — AA, AL, ATTR, and Abeta2m — is based on the protein deposited in the tissues. The first letter, A, stands for “amyloidosis.” The subsequent letter(s) indicate(s) the type of protein involved. For example, in AA amyloidosis, the second letter A stands for serum amyloid A protein; in AL amyloidosis, the letter L stands for (immunoglobulin) light chain; in ATTR amyloidosis, TTR stands for “transthyretin.” [2, 4].

The incidence of new cases of amyloidosis, especially ATTR and AL amyloidosis, has been increasing in recent years in association with the aging of the population and greater access to diagnostic tests, including research of genetic mutations related to the disease, better cardiac imaging for recognition of cardiac involvement (i.e., magnetic resonance imaging, pyrophosphate myocardial scintigraphy, and echocardiography), and use of mass spectroscopy, immunohistochemistry, and electron microscopy for improved characterization of the type of amyloid protein involved in the disease. [6, 7] Conversely, the prevalence of rheumatologic disorders in AA amyloidosis derived from chronic inflammatory conditions (also known as secondary or reactive amyloidosis) is progressively decreasing, particularly in developed countries, due to the introduction of effective new treatments. [2, 3, 8, 9]

From a clinical perspective, systemic amyloidosis often involves the kidneys (causing nephrotic syndrome), the heart (cardiac failure and arrhythmia), and the peripheral nervous system (sensorimotor polyneuropathy and autonomic dysfunction). Increased organ size from extracellular amyloid deposits, with liver and spleen enlargement, is a frequent finding. Gastrointestinal complications with diarrhea and bleeding also occur. Additionally, musculoskeletal involvement with arthralgias and subchondral cystic bone lesions, as well as carpal tunnel syndrome, may be present. [2, 3] (Table 1).

**Table 1:** Most common types of amyloidosis, clinical findings, and treatment

	AA	AL	ATTRv	ATTRwt	AB2M
Protein that causes amyloid deposits	serum amyloid A (SAA) protein	kappa or lambda light chains	mutated transthyretin misfolding	age-related transthyretin misfolding	beta2-microglobulin
Clinical features	renal involvement with edema, isolated proteinuria, and/or renal failure	heart failure, spontaneous ecchymosis and periorbital purpura, macroglossia, soft-tissue infiltration in the shoulders, bilateral carpal tunnel syndrome and GI involvement	polyneuropathy, autonomic dysfunction, heart failure, cardiac arrhythmias, bilateral carpal tunnel syndrome	heart failure, autonomic dysfunction, bilateral carpal tunnel syndrome, acquired lumbar spinal stenosis, and rupture of the long biceps tendon	cystic bone lesions, involvement of the cervical spine, wrists, and shoulders, and infiltration of musculoskeletal soft tissues with bilateral carpal tunnel syndrome
Treatment	biological agents- anti-TNF, anti-IL6 and anti-IL1	Hematopoietic transplantation, combination of chemotherapy, steroids, bortezomib, daratumumab	transthyretin stabilizers, oligonucleotide therapy, liver transplantation	transthyretin stabilizers	hemodialysis with high-flux biocompatible membranes, which remove beta2-microglobulins effectively, and dialysates that minimize inflammation and production of beta2-microglobulin

*Legend* AA - A amyloidosis; AB2M - beta2-microglobulin amyloidosis; AL - light chain amyloidosis; ATTRv - hereditary amyloidogenic transthyretin amyloidosis with polyneuropathy; ATTRwt - wild-type transthyretin amyloidosis; GI - gastrointestinal; IL1 - interleukin 1; IL6 - interleukin 6; TNF - tumor necrosis factor

Amyloidosis has different clinical presentations with many overlapping manifestations, independently on the type of amyloid protein involved. Identifying the type of amyloidosis is fundamental, as a correct clinical suspicion will translate into diagnostic tests and therapeutic approaches relevant to the suspected diagnosis. [2–4] The clinical diagnosis of primary and secondary amyloidosis requires a comprehensive and systematic approach, involving a combination of clinical assessment, laboratory tests, imaging studies, and histopathological examination. The initial clinical suspicion of primary amyloidosis often arises from nonspecific symptoms such as fatigue, weight loss, and edema, coupled with organ-specific manifestations like nephrotic syndrome, cardiomyopathy, hepatomegaly, or neuropathy. On the other hand, the diagnostic process for AA secondary amyloidosis similarly begins with clinical suspicion based on chronic inflammatory disease history and symptoms like renal dysfunction, hepatosplenomegaly, and gastrointestinal involvement.

**Fig. 1:**
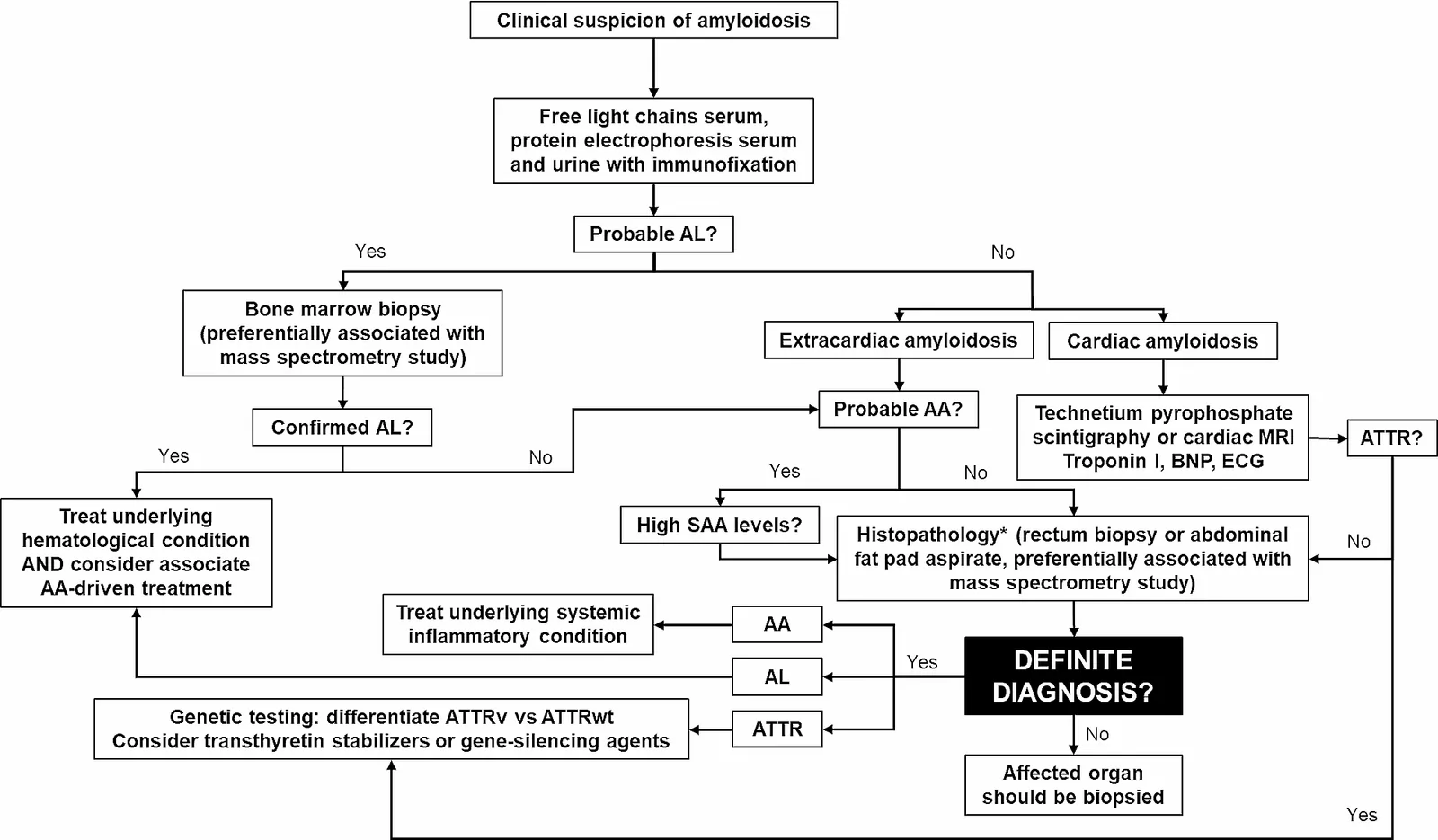
Workflow for diagnosis of the type of amyloidosis in case of suspicion. *Legend* AA - A amyloidosis; AL - light chain amyloidosis; ATTRv - hereditary transthyretin amyloidosis ATTRwt - transthyretin amyloidosis wild type; BNP - brain natriuretic peptide; ECG – eletrochardiogram; SAA - serum A amyloid protein. * Abdominal fat pad aspirate or rectum biopsy in ATTR may not be necessary if other complementary tests have already provided a definite diagnosis

In both conditions, a key diagnostic step still involves confirmation through tissue biopsy, typically from an easily accessible site such as abdominal fat, the rectum, or the affected organ, with Congo red staining demonstrating apple-green birefringence under polarized light, indicative of amyloid deposits. Mass spectrometry or immunohistochemistry is subsequently employed to determine the amyloid type, crucial for directing therapy. Algorithms for the diagnostic evaluation of amyloidosis typically emphasize a stepwise approach, starting with clinical and laboratory screening, followed by targeted imaging and definitive histopathological confirmation (Fig. 1). This structured methodology not only facilitates accurate diagnosis but also informs prognosis and guides therapeutic decisions, highlighting the critical importance of early and precise identification of amyloid type in optimizing patient outcomes.

Based on these considerations, the aim of the present review is to discuss the clinical characteristics of different types of amyloidosis to facilitate their identification by rheumatologists.

## Method

This is a narrative review in which the authors synthesize and discuss the updated and general knowledge about systemic amyloidosis based on a random search of the medical literature in a non-systematic way. The main articles published in english extracted from the database medline (OVID) were reviewed with the terms systemic amyloidosis, hereditary amyloidosis, secondary amyloidosis, cardiac amyloidosis and musculoskeletal or rheumatic manifestations of amyloidosis, as well as the different types of treatments. The references included in a non-systematic way resulted in the lack of hierarchy in the selected evidence. The authors confirm that limitations with this review may arise due to the non-systematized selection of literature with the sporadic search for articles.

### AA (secondary or reactive) amyloidosis

AA amyloidosis occurs when the liver produces excessive insoluble serum amyloid A (SAA) protein upon stimulation by proinflammatory cytokines in the setting of undiagnosed (or untreated or uncontrolled) chronic systemic inflammatory diseases. Although obsolete, herein we preferred keeping the term “secondary” for this subtype of amyloidosis in an attempt of calling the attention that it is usually caused by an underlying systemic inflammatory disease. Conditions that cause AA amyloidosis include immune-mediated diseases like rheumatoid arthritis (RA), spondyloarthritis (SPA), inflammatory bowel diseases, and autoinflammatory diseases, among others. In addition, chronic infections like tuberculosis, leprosy, chronic osteomyelitis, and bronchiectasis are still an important etiology of AA amyloidosis in developing countries and an emerging cause in developed countries. [8, 9] Independent from the underlying disease, patients with AA amyloidosis have *SAA1* alleles that lead SAA proteins to aggregate as amyloid fibrils and interfere with their susceptibility to degradation by matrix metalloproteinases. [10–12]

AA amyloidosis currently accounts for about 10% of all cases of amyloidosis, with equal distribution between men and women. [9] In most cases, AA amyloidosis leads to renal impairment with edema, isolated proteinuria, and/or renal failure; in these patients, chronic renal disease occurs with kidneys of normal or enlarged size. [9] Bowel involvement may occur in patients with AA amyloidosis, leading to malabsorption, diarrhea, weight loss, or gastrointestinal bleeding. Cardiac involvement is rare in this form of the disease and typically occurs late in its course. In these cases, monitoring with biomarkers (troponin I, brain natriuretic peptide [BNP], or N-terminal prohormone of BNP [NT-proBNP]) is usually unable to predict future cardiac involvement. [13] Of note, the polyneuropathy that occurs in other types of amyloidosis (e.g., AL and hereditary ATTR) is not present in AA amyloidosis. [9, 13–15]

The diagnosis of AA amyloidosis is based on biopsy of involved organs and tissues, including minor salivary glands (lips), abdominal fat pad, and kidneys. For the initial biopsy, tissues that are easily accessible are preferable over those of more difficult access (e.g., kidneys or liver), as they involve less invasive procedures with a lower chance of bleeding.

Fat punch biopsy yields more material for analysis than fat aspiration. In a prospective study, ultrasound-guided percutaneous core needle biopsy of subcutaneous fat had a sensitivity and specificity of 85.7% and 100%, respectively. Minor salivary gland biopsy is also sensitive (68.4%) in detecting AA amyloid. [16, 17]

AA amyloidosis must be documented histologically since other immune-mediated diseases may also play a role in the differential diagnosis. Immunohistochemistry using antibodies against the SAA protein confirms the diagnosis. [9, 13]

Tests to exclude AL amyloidosis must be initially carried out in patients with probable AA amyloidosis. The workup should include free light chain test and serum and 24-hour urine protein immunofixation. Levels of SAA protein can be measured to confirm increased production and monitor treatment. [9, 13] The evaluation should be done during the intercritical period, especially in autoinflammatory diseases, as this biomarker is expected to be elevated during flares. Additionally, normal SAA levels do not exclude the possibility of AA amyloidosis in certain autoinflammatory diseases or chronic infections.

Early diagnosis and treatment of AA amyloidosis is fundamental, as this condition is associated with decreased survival (between 6 and 9 years). Nevertheless, overall survival in AA amyloidosis is significantly better than that observed in AL and ATTR amyloidosis. [14, 18] The prognosis of AA amyloidosis is influenced by several critical factors that collectively shape the assessment and management strategies tailored to individuals with this condition, highlighting the complexity and diverse clinical presentations of the disease. Renal function assumes a pivotal role, as initial renal failure at diagnosis significantly predicts progression to end-stage renal disease, despite effective management of the underlying inflammatory process. Digestive symptoms such as diarrhea, malabsorption, bleeding, or perforation can pose life-threatening challenges in some cases. The effectiveness of anti-inflammatory therapies in managing and mitigating inflammation also plays a significant role in determining prognosis. Furthermore, comorbidities, particularly atherosclerosis exacerbated by chronic inflammation, progress more rapidly in patients undergoing hemodialysis, thereby presenting additional complexities in prognostic assessment and management. [19]

### Transthyretin amyloidosis

The tetrameric protein TTR is synthesized primarily in the liver and is responsible for transporting vitamin A and thyroxine. ATTR amyloidosis may be hereditary (ATTRv) or of late onset or wild type (ATTRwt). ATTRv is caused by a genetic mutation in the *TTR* gene, resulting in abnormal transthyretin protein that builds up as an insoluble fibrillar form. This form of amyloidosis has an autosomal dominant transmission with variable penetrance.

ATTR amyloidosis accounts for 10–20% of the cases in referral centers. Notably, the incidence of non-hereditary ATTRwt is on the rise. The early appearance of ATTRv and its main involvement of peripheral nerves, causing familial amyloidotic polyneuropathy, garner particular attention in these cases. [2, 3] The variant most commonly implicated in this phenotype is p.Val50Met.

The manifestations of ATTRv include dry eyes causing conjunctivitis, hepatomegaly in cases of advanced-stage disease, gastrointestinal symptoms with diarrhea or constipation, nausea and vomiting, bilateral carpal tunnel syndrome, and purpura in advanced stages. The classical peripheral neuropathy in this hereditary form of amyloidosis is often associated with autonomic dysfunction. [2, 3]

ATTRwt, an acquired form of amyloidosis, is more common in the male sex and is associated with musculoskeletal manifestations that must be identified, including bilateral carpal tunnel syndrome, acquired lumbar spinal stenosis, and rupture of the long biceps tendon. Lumbar spinal stenosis results from amyloid deposits in the ligamentum flavum in the lumbar spine. [2, 3, 20, 21]

A possible tool for diagnosing ATTR is an abdominal fat pad fine-needle aspiration biopsy. Although it is a noninvasive procedure, it has low sensitivity, around 15% in wild-type ATTR. Additionally, a negative fat biopsy result does not rule out ATTR amyloidosis. Additional approaches, such as cardiac scintigraphy, genetic testing, or myocardial biopsy, should also be considered in these cases [22]. 

Cardiac involvement is the main determinant of increased mortality in ATTR amyloidosis. The median survival after diagnosis in untreated patients is very short (2.5 years in ATTRv and 3.6 years in ATTRwt). The occurrence of HF is less frequent and less severe in ATTRv amyloidosis compared with ATTRwt and AL amyloidosis. [23–26]

Risk factors for worse prognosis include specific clinical features (worse performance on functional assessments, specific genotypes, and comorbidities such as renal dysfunction), increased myocardial stress evidenced by elevated biomarkers (troponin and NTproBNP), worsening pump function visualized via echocardiography (left ventricular ejection fractions, myocardial contraction fraction, stroke volume index or global strain/strain patterns), and the magnitude and pattern of amyloid infiltration visualized via uptake on nuclear scans (heart to contralateral ratio) or via cardiac magnetic resonance (late gadolinium enhancement and extracellular volume). [27]

### AL amyloidosis

AL amyloidosis is derived from amyloidogenic immunoglobulin light chains. In this disease type, kappa or lambda light chains, or fragments of these monoclonal light chains, are deposited in organs in a fibrillar form. Organ dysfunction in AL amyloidosis is believed to result not only from tissue deposits but also from a direct toxic effect. [28] The number of cases of AL amyloidosis has remained stable, and this type of amyloidosis accounts for most of the current cases of amyloidosis (about 67%). [7]

Different monoclonal immunoglobulin-secreting conditions may be associated with AL amyloidosis, including monoclonal gammopathy of unknown significance, non-lymphoplasmacytic lymphoma, multiple myeloma, lymphoplasmacytic lymphoma (Waldenström’s macroglobulinemia), mucosa-associated lymphoid tissue lymphoma, and chronic lymphocytic leukemia. [7, 29] According to a study, the average time from symptom onset to diagnosis in AL amyloidosis is 2.7 years, and patients often consult different doctors until the diagnosis is finally established. [30]

Multiple myeloma is present in approximately 10% of patients with AL amyloidosis, and leads to the classic findings of hypercalcemia, renal failure, anemia, and osteolytic bone lesions. [31, 32]

About 60–70% of patients with AL amyloidosis have the classic characteristics of renal involvement with nephrotic syndrome. [33] The heart is the second most affected organ, with severe heart failure (HF) being the main determinant of survival. [6, 7]

Classic findings specific to AL amyloidosis include the presence of spontaneous ecchymosis and periorbital purpura (Racoon’s eyes) attributed to increased amyloid deposition in small blood vessels, X factor deficiency, macroglossia, soft-tissue infiltration in the shoulders with local increased volume causing pseudohypertrophy, and bilateral carpal tunnel syndrome. Autonomic nervous system dysfunction can lead to gastrointestinal symptoms, manifesting with diarrhea, weight loss, and gastrointestinal bleeding, is also characteristic of this form of amyloidosis. Approximately 20% of patients have sensorimotor polyneuropathy. [7]

The presence of proteinuria in a patient with monoclonal gammopathy should raise the suspicion of amyloidosis. [34] Amyloid infiltration into blood vessels causes claudication of the jaw or lower limbs. Amyloid deposition in AL amyloidosis also causes nail dystrophy and alopecia. [7]

The sensitivity of serum protein electrophoresis in diagnosing AL amyloidosis is 70%; the sensitivity increases to > 90% with serum immunofixation and exceeds 99% when urine protein immunofixation and free light chain tests are added. [35, 36] In the absence of monoclonal light chains, bone marrow biopsy with immunohistochemistry and FISH should be performed in AL amyloidosis for the characterization of the B-cell clone before starting the specific treatment. Screening for bone lesions should be performed in patients with a concomitant multiple myeloma. An alternative option is a fine-needle aspiration biopsy of the abdominal fat pad. This method offers excellent specificity and positive predictive value (both 100%), but it has lower sensitivity, which is around 84% for AL amyloidosis, and there is a significant rate of inadequate specimens (11%). [37]

The management of AL amyloidosis depends on staging and prognostication. In 2012, the Mayo Clinic updated their staging system to include the dFLC (difference between involved and uninvolved free light chains), in which levels greater than 18 mg/dL are considered significant and revised the cardiac biomarkers to NT-proBNP (> 1800ng/L) and cardiac TnT (> 0.025mcg/L). [38] Stage I includes patients with no elevated risk factors, while stages II, III, and IV encompass those with one, two, or three elevated risk factors, respectively. These staging criteria help guiding treatment decisions and prognostication in AL amyloidosis management. [39]

### Dialysis-related or beta2-microglobulin amyloidosis

This rare subtype of amyloidosis occurs in patients with chronic kidney disease who have been on long-term hemodialysis. It may also affect patients undergoing peritoneal dialysis and those with chronic kidney disease even before dialysis. [40] The pathophysiology of beta2-microglobulin amyloidosis (AB2M) involves the accumulation and deposition of β2M amyloid fibrils in various tissues, particularly the osteoarticular structures. β2M is a component of the major histocompatibility complex class I molecules, and under normal renal function, it is efficiently cleared by the kidneys. However, in chronic kidney disease patients, impaired renal clearance leads to elevated serum β2M levels. During dialysis, β2M is inadequately removed due to its high molecular weight, resulting in its systemic accumulation. Over time, β2M undergoes conformational changes, leading to the formation of insoluble amyloid fibrils. The pathogenesis is further exacerbated by oxidative stress, inflammation, and advanced glycation end products.

These fibrils deposit in synovial membranes, tendons, and bones, causing a spectrum of clinical manifestations, including cystic bone lesions, involvement of the cervical spine, wrists, and shoulders, and infiltration not only of musculoskeletal soft tissues, with bilateral carpal tunnel syndrome, but also synovia, causing destructive arthropathy. Cardiac involvement is rare in this form of amyloidosis. [41]

The clinical prognosis is generally poor, with outcomes closely linked to the duration of dialysis and the extent of β2M amyloid deposition. Prognostic stratification involves assessing both the severity and distribution of amyloid deposits and their impact on patient’s functional status and quality of life. High-risk patients often exhibit extensive osteoarticular involvement and recurrent infections due to amyloid-related immune dysfunction. Two technological improvements introduced to prevent the occurrence and mitigate the effects of amyloidosis related to hemodialysis include high-flux biocompatible membranes, which remove beta2-microglobulins effectively, and dialysates that minimize inflammation and production of beta2-microglobulin. [41] Despite these interventions, AB2M remains a challenging condition with a significant impact on morbidity and mortality, underscoring the need for early detection and personalized management strategies.

### Cardiac involvement in AL and ATTR amyloidosis

In the initial stages, HF in ATTRwt and AL amyloidosis has the classic presentations of restrictive cardiomyopathy with preserved ejection fraction. With progression, there is an important increase in left ventricular wall thickness (sparing the heart apex and occurring in the absence of hypertension) and involvement of the right ventricle and atria. There is a noticeable discrepancy between ventricular mass and electrocardiogram, in which small, low QRS voltages and a pseudo-infarct pattern may be present. Arrhythmias also occur, with atrioventricular conduction abnormalities and atrial tachyarrhythmias. [6]More frequently observed in ATTR than in AL amyloidosis, low-flow, low-gradient severe aortic stenosis may also be present. In patients with HF, the concomitant occurrence of syncope, autonomic dysfunction, and peripheral neuropathy and the presence of a cardiac pacemaker should draw attention to cardiac amyloidosis. Other findings include persistently elevated biomarkers (troponin I, NT-proBNP, or BNP) in disproportion to the degree of HF. Unexpected resolution of hypertension, reduced tolerance to antihypertensive drugs, and progressive reduction in the number and doses of antihypertensive drugs should also draw attention to the occurrence of cardiac amyloidosis. [6]Invasive procedures (e.g., endomyocardial biopsy) to confirm the diagnosis of cardiac amyloidosis are not required in most cases, as disease-specific findings may be present on cardiac magnetic resonance imaging, myocardial scintigraphy, and echocardiogram. [6, 14] It should be performed if other infiltrative diseases such as Fabry disease, sarcoidosis, and histiocytosis have not been ruled out, or if noninvasive procedures have not provided clarification. Increased myocardial uptake of technetium pyrophosphate on cardiac scintigraphy, although not widely available, may suggest cardiac amyloidosis due to ATTR, particularly ATTRwt, but may also indicate AL amyloidosis. [6] When cardiac amyloidosis is suspected, algorithms suggest the exclusion of AL amyloidosis as the initial step; if AL amyloidosis is excluded, myocardial scintigraphy with pyrophosphate should be obtained to confirm the diagnosis of ATTR [6] and genetic tests should be conducted (Fig. 1).

### Treatment

#### AA amyloidosis

In AA amyloidosis, treatment is directed toward the underlying disease. Decreases in SAA protein levels slow the progression of the disease and are associated with longer survival. [13, 42–44]Although limited to a few case reports given the rarity of this type of amyloidosis, the clinical response regarding renal outcomes appears favorable, especially with tumor necrosis factor (TNF) and interleukin 6 (IL-6) inhibitors. [13, 45–50] In patients with AA amyloidosis requiring kidney transplant due to renal function loss, the chance of recurrence of amyloidosis in the transplanted kidney is very low once good control of the underlying inflammatory disease is achieved. [51] Of note, the early use of colchicine in patients with familial Mediterranean fever has been associated with prevention of AA amyloidosis. [52] Moreover, antibiotics may be used for recurrent infections, and surgery may be considered for monocentric Castleman disease.

#### Transthyretin amyloidosis

Since the mutated transthyretin protein is produced in the liver, liver transplant was attempted initially as a treatment option in patients with ATTRv, but neuropathy and cardiomyopathy continued to progress in some patients. Current therapies for ATTRv include transthyretin stabilizers, which maintain the transthyretin form limiting the rate of transthyretin dissociation into monomers and consequent amyloid formation (e.g., diflunisal, tafamidis, and acoramidis, a.k.a. AG10), and gene-silencing agents, which modulate gene expression to reduce hepatic transthyretin synthesis(e.g., patisiran and inotersen). The efficacy of inoserten in ATTR cardiomyopathy is currently being evaluated. [23, 53] Other agents, such as eprodisate, disrupt preexisting amyloid fibrils and promote their removal by macrophages. [53]

Tafamidis is the first FDA-approved drug for treating cardiomyopathy associated with ATTRv or ATTRwt, as it stabilizes cardiac dysfunction and polyneuropathy in combination with doxycycline and tauroursodeoxycholic acid. Acoramidis is currently being evaluated in clinical trials for treating ATTRv and ATTRwt cardiomyopathy. Additionally, tolcapone — an FDA-approved medication for Parkinson’s disease — has been shown to stabilize transthyretin. [4] Employing CRISPR/Cas9 technology (NTLA-2001), the TTR gene can be knocked out in patients with ATTR amyloidosis with just a single administration. In a Phase I study involving a small cohort of patients with ATTRv amyloidosis and polyneuropathy, NTLA-2001 administration reduced serum TTR protein levels. [54]

AL amyloidosis.

Treatment in AL amyloidosis is directed at reducing light chain production by the bone marrow and includes chemotherapy and stem cell transplantation. [55, 56] However, criteria for selecting patients for treatment with autologous stem cell transplantation are strict, as confirmed tissue diagnosis of AL amyloidosis with end organ damage and minimum performance status (Eastern Cooperative Oncology Group 0–2) are required, including age of 18–70 years, left ventricular ejection fraction > 40%, oxygen saturation > 95% in room air, diffusing capacity of the lungs for carbon monoxide > 50% of predicted, absence of medically refractory pleural effusions, direct bilirubin < 2 mg/dL, estimated glomerular filtration rate > 30 ml/min/1.73 m², and baseline supine systolic blood pressure > 90mmHg. [57] Therefore, most patients are typically treated with a combination of chemotherapy, steroids, and bortezomib. [7, 58]

Monoclonal antibodies such as daratumumab (anti-CD38) and belantamab mafodotin (anti-B cell maturation antigen) emerged as a safe and effective therapeutic option both as monotherapy or combined with standard treatments. [59] Since 2021, studies on the treatment of AL amyloidosis using CAR (chimeric antigen receptor) T cell therapy have also emerged. [60, 61] The advantages of using these therapies in AL amyloidosis include a low plasma cell burden and the potential for achieving durable responses. Nevertheless, challenges such as cytokine release syndrome still need to be addressed in this high risk population. [57]

The International Society of Amyloidosis has established response criteria for AL amyloidosis, which have recently been clarified. Complete response requires the absence of amyloidogenic light chains (either free or as part of a complete immunoglobulin) as determined by negative immunofixation electrophoresis of both serum and urine, along with either a free light chain (FLC) ratio within the reference range or the uninvolved FLC concentration greater than the involved FLC concentration, regardless of an abnormal FLC ratio. A very good partial response is characterized by a difference in dFLC concentration of less than 40 mg/L. For a partial response, there must be a greater than 50% decrease in dFLC concentration compared to baseline. Patients who do not meet any of these criteria are classified as having no response. [27]

## Conclusions

Amyloidosis is a group of diseases caused by several proteins that determine different clinical presentation and needs to be diagnosed early. This condition interest rheumatologists due to its systemic and musculoskeletal involvement and its association with rheumatic diseases. New diagnostic tools such as mass spectroscopy, immunohistochemistry and cardiac imaging tests contribute to earlier recognition. New treatments for both AA, AL and ATTR forms of amyloidosis, with reduced mortality and better outcomes justify more interest in this condition.

## Data Availability

Data sharing not applicable – no new data generated.
